# Unusual Form of Heart Failure!

**DOI:** 10.5935/abc.20160062

**Published:** 2016-07

**Authors:** Rita Ferreira, Silvia Monteiro, Mariano Pego

**Affiliations:** Centro Hospitalar e Universitario de Coimbra - Hospitais da Universidade de Coimbra, Coimbra - Portugal

**Keywords:** Pulmonary Edema, Hernia, Hiatal / surgery

**Figure 1:**
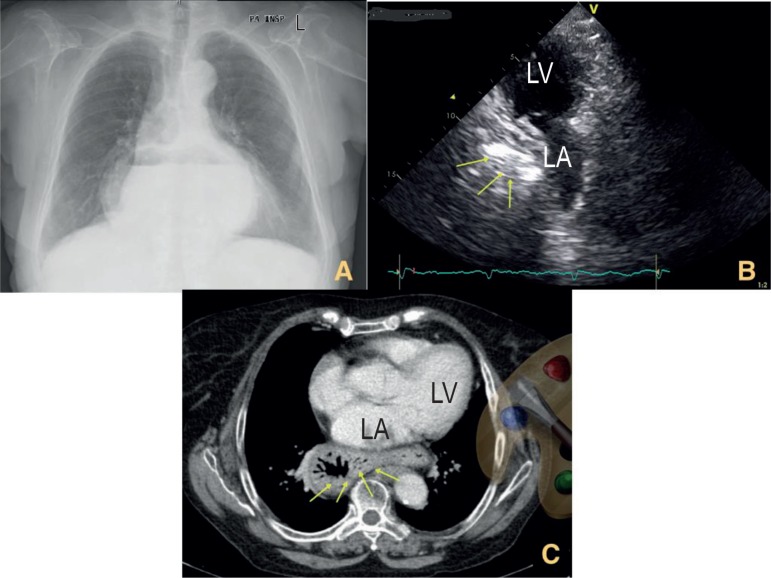
A) The chest radiography showed congested lung and a round shadow behind the heart; B) Transthoracic echocardiography showing echolucent mass severely compressing the left atrium; C) Spiral computed tomography of thorax showed a large hiatus hernia with intrathoracic extension.

An 80-year old woman was admitted to the emergency room due to severe dyspnea. She had no known history of cardiovascular disease, but in last month she complained to increasing fatigue and shortness of breath. Physical examination was notable for diffuse lungs crackles. The heart sounds were normal and no murmur could be heard. The 12-lead electrocardiogram demonstrated sinus rhythm with non-specific ST-segment changes. The chest radiograph showed congested lung and there was also a round shadow behind the heart (figure 1-A). The patient was admitted to Cardiology department for further investigation.

In the next day, a two-dimension transthoracic echocardiography was performed and showed normal left ventricular function, no valvular disease, but the left atrium was severely compressed by an extrinsic amorphous, echolucent mass (Figure 1-B). Spiral computed tomography of thorax showed a large hiatus hernia with intrathoracic extension (Figure 1-C). The hernia was located behind the left atrium causing anterior shift of the heart. The intrathoracic migration of a large part of the stomach was confirmed by upper gastrointestinal barium examination, which was performed after consulting a surgeon, to further assess the extent of the hernia and the potential need for surgical treatment.

In the first few months, the patient was successfully treated with conservative measures, but the symptoms returned and she was admitted again with acute pulmonary edema. One week later, the corrective surgery was performed and the patient had no further recurrence of acute pulmonary edema in the subsequent 6 months.

In the light of these knowledges, FFR might be a useful tool to evaluate moderate coronary lesions with regard to revascularization appropriateness.

